# Genomics Reveals Distinct Evolutionary Lineages in Asian Elephants

**DOI:** 10.1002/ece3.72019

**Published:** 2025-08-18

**Authors:** Jeroen Kappelhof, Emma Diepeveen, Martijn F. L. Derks, Ole Madsen, Rebekah Rogers, Benoit Goossens, Reeta Sharma, Martien A. M. Groenen, Jack J. Windig, Mirte Bosse

**Affiliations:** ^1^ Animal Breeding and Genomics Wageningen University and Research Wageningen the Netherlands; ^2^ Royal Rotterdam Zoological & Botanical Gardens Rotterdam the Netherlands; ^3^ A‐LIFE, Section Ecology & Evolution Vrije Universiteit Amsterdam Amsterdam the Netherlands; ^4^ Department of Bioinformatics and Genomics University of North Carolina Charlotte North Carolina USA; ^5^ Danau Girang Field Centre C/o Sabah Wildlife Department Kota Kinabalu Sabah Malaysia; ^6^ Organisms and Environment Division, Cardiff School of Biosciences Cardiff University Cardiff UK; ^7^ Wildlife Institute of India Dehradun India

## Abstract

This study introduces, for the first time, whole‐genome sequencing (WGS) data from predominantly wild‐born Asian elephants currently housed in European zoos, covering the distribution range of Asian elephants. With this WGS data, we aim to validate the current designation of Asian elephant subspecies and address currently discussed ambiguities about their origin, particularly concerning Bornean and Sri Lankan elephants by analyzing population structure, determining divergence times, and exploring ancient and recent bottlenecks. Understanding the evolutionary history of the Asian elephant subspecies is essential for developing targeted conservation strategies and mitigating risks to their survival. Analysis reveals a clear population structure with relatively recent splits, delineating three distinct genetic clusters: Borneo, Sumatra, and Asian Mainland, with Sri Lanka forming an additional group. We estimated the divergence time between Bornean and Sumatran elephants to be around 170,000 years ago. The divergence of the Sri Lankan elephant from the Mainland is estimated to have occurred around 48,000 years ago, with Sri Lankan elephants predominantly clustering with those from Myanmar, possibly due to historical trade networks. The genome of the Bornean elephant exhibited signatures of severe bottlenecks as recently as 8 and 38 generations ago, further supporting hypotheses of their introduction. Our data reflect the current Asian elephant subspecies designation. Additionally, for the first time, the Sumatra elephant is confirmed as a distinct subspecies with genomic data. Furthermore, the study discusses genetic management strategies for ex‐situ populations, emphasizing the importance of implementing cluster‐specific conservation measures.

## Graphical abstract

This study introduces, for the first time, whole‐genome sequencing (WGS) data from predominantly wild‐origin Asian elephants housed in European zoos, covering the distribution range of Asian elephants. The WGS data aim to validate the Asian elephant subspecies designation and address ambiguities about their origin, particularly concerning Bornean and Sri Lankan elephants.

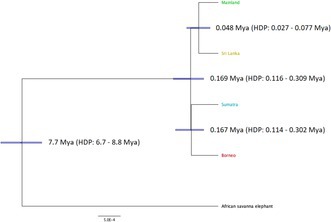

## 1 Introduction

The Asian elephant ( *Elephas maximus* ), a keystone species, faces a critical juncture as its populations decline due to a myriad of threats including habitat fragmentation, human‐elephant conflict, and poaching (Leimgruber et al. 2003; Sukumar 2003; Menon and Tiwari 2019). This decline has left the species restricted to fragmented and isolated pockets across South and South‐East Asia, erasing its presence from West Asia, Java, and significant parts of China (Menon and Tiwari 2019; Fernando and Lande 2000).

Currently, the global free‐ranging population of Asian elephants is estimated at around 50,000 individuals (Menon and Tiwari 2019). Notably, India is home to nearly 60% of the global free‐ranging population, highlighting its significance in elephant conservation (Menon and Tiwari 2019). The International Union for Conservation of Nature (IUCN) Red List categorizes the global status of Asian elephants and the Bornean elephant subspecies as Endangered, while the Sumatran elephant subspecies is assigned as Critically Endangered (Williams et al. 2020; IUCN 2024).

Central to conservation efforts is an understanding of the taxonomic complexities within the Asian elephant population. Presently, four subspecies are recognized by the International Union for Conservation of Nature (IUCN) Red List: *E. maximus indicus* on the mainland, *E. maximus maximus* in Sri Lanka, *E. maximus sumatranus* on Sumatra, and *E. maximus borneensis* on Borneo (Williams et al. 2020; IUCN 2024). These designations stem from a combination of morphological distinctions and genetic markers, notably mitochondrial DNA (mtDNA) haplotypes (Fernando et al. 2000). Some molecular genetic studies using mtDNA indicate no major genetic differentiation between the Sri Lankan elephant and the mainland populations (Hartl et al. 1996; Fernando and Lande 2000; Fleischer et al. 2001; Sukumar 2003). Therefore, it has been suggested that *E. maximus indicus* and *E. maximus maximus* should not be considered distinct subspecies. However, for species with male‐mediated gene flow, as is the case for Asian elephants, caution needs to be taken when conclusions are drawn based on mtDNA analyses, because any persistent mtDNA geographic partitioning may not reflect nuclear population structure (Ishida et al. 2011).

Additionally, the classification of *E. maximus borneensis* on Borneo as an Asian elephant subspecies has been a subject of debate for a long time, while they are now recognized as an Asian elephant subspecies (IUCN 2024). Two contradicting hypotheses about their origin have been proposed: one stating that elephants are not native to Borneo and have been introduced by humans between the 16th and 18th centuries; the other stating that elephants have colonized the island during Pleistocene glaciations and are thus native to Borneo (Fernando et al. 2003; Sharma et al. 2018). The introduction hypothesis is based on historical records suggesting that the current population on Borneo represents the descendants of a domesticated herd that formerly existed on Sulu Island, Philippines, and was introduced to Eastern Sabah by the Sultan of Sulu (Earl of Cranbrook et al. 2008; Shim 2003; Sharma et al. 2018). The now extinct Javan elephant population is suggested as the source population for this domesticated herd, while other populations from the mainland have also been suggested to be the source (Sharma et al. 2018). Currently, genomic data from Javan elephants is not available, which makes it difficult to draw definite conclusions.

Genomics has proven a pivotal tool in addressing taxonomic uncertainties (Feder et al. 2012). The genetic makeup of populations provides insights into their evolutionary relationships and divergence patterns. However, unraveling these complexities requires the integration of various factors, including migration, isolation, and historical events (Ravinet et al. 2017). Genetic diversity can be shaped by both inbreeding and outbreeding phenomena, which can impact the fitness of populations. The challenge lies in keeping a balance that ensures the long‐term viability of these subspecies and their potential evolutionary significant units (ESUs).

Whole‐genome sequencing (WGS) has emerged as a potent technique for disentangling genetic intricacies, such as complex patterns of relatedness and mosaic ancestry, because of its high resolution and the coverage of the whole genome (Raphael et al. 2021; Sun et al. 2023). While WGS data for a few Asian elephant populations are accessible for places like Borneo, India, and Myanmar, gaps exist in data availability for populations like Thailand, Vietnam, and Sumatra (Tollis et al. 2021). Furthermore, the uneven amount of WGS data available from the different Asian elephant populations adds complexity to accurate population structure analyses.

**FIGURE 1:**
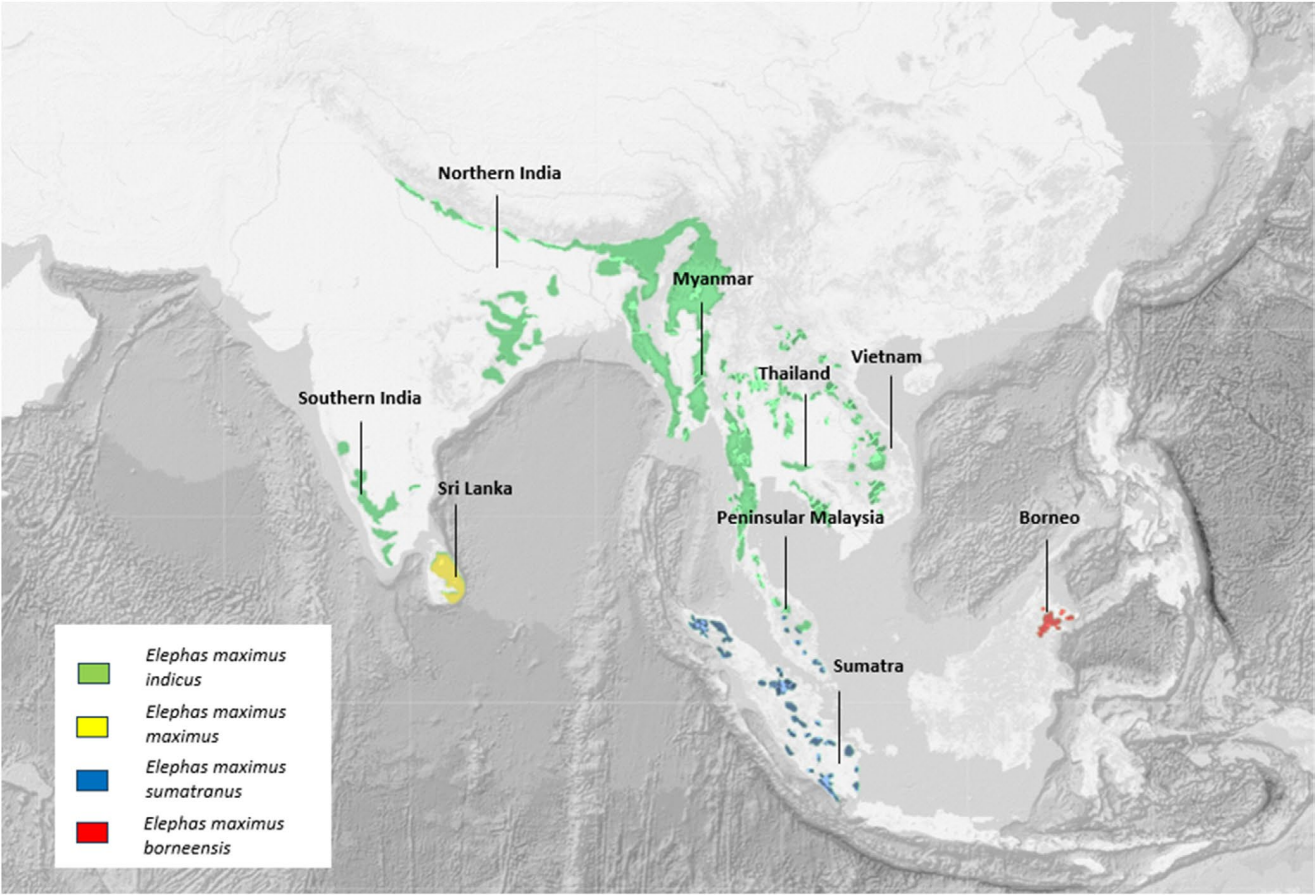
Current Asian elephant subspecies designation and Asian elephant populations included in this study. The origin of samples is at the country level, so exact geographical location is unknown.

For this study we assembled WGS data from 27 wild‐born Asian elephants or offspring from two wild‐born Asian elephants covering almost the entire distribution range of Asian elephants (Figure 1) and provide new insights into the phylogeny of Asian elephants. We aim to validate the current Asian elephant subspecies designation with WGS data, primarily concerning the Sri Lankan and Bornean elephants, which will contribute to the optimization of in situ and ex situ conservation strategies for this species. For this, we will (1) analyze population structure, (2) determine divergence times between the four Asian elephant subspecies, and (3): explore ancient and recent bottlenecks. Taking into account the current designation of Asian elephant subspecies, we expect to identify four genetic clusters within the Asian elephant population. Furthermore, based on previous studies, we expect relatively recent divergence times, especially in comparison to African elephants. Moreover, we expect to find both ancient and recent bottlenecks within Asian elephants caused by ancient ice ages and recent anthropogenic influences. Especially for the Bornean elephant we expect to find recent bottlenecks due to their high levels of inbreeding.

## 2 Material and Methods

An overview of software used for analysis is listed in Table S1.

### 2.1 Sample and Data Collection

Blood samples of 23 individual Asian elephants located in zoos that are members of the European Association of Zoos and Aquaria (EAZA) were provided by the EAZA Biobank (Table S2). In all cases, these blood samples were retrieved during health check‐ups or regular training sessions. Individuals for sampling were chosen based on the availability and accuracy of data about their wild origin. Data about their origin was retrieved via the EAZA Ex situ Program (EEP) coordinator for Asian elephants. This data is registered within Species360 Zoological Information Management System (ZIMS; https://zims.species360.org/). Moreover, we intended to represent each assigned subspecies and maximize the coverage over the geographical distribution range of the Asian elephant. DNA extraction was done with the Puregene blood kit of Qiagen with associated protocol ‘DNA purification from whole blood’. The DNA samples were sent to BGI TECH SOLUTIONS and Novogene and sequenced to a depth of ~25× using Illumina Novaseq Sequencing resulting in 150 bp paired end reads. Raw short read sequences of 4 Asian elephant individuals were obtained from public online repositories (Wilkie et al. 2013; Tollis et al. 2021). Our dataset included 27 Asian elephant individuals of which 18 individuals originate from the mainland (Northern India *n* = 3, Southern India *n* = 3, Myanmar *n* = 3, Thailand *n* = 3, Vietnam *n* = 3, Peninsular Malaysia *n* = 3), three individuals from Sri Lanka, three individuals from Sumatra and three individuals from Borneo. For phylogenetic analysis, one African elephant genome ( *Loxodonta africana* ; sample: ERR2260497) was retrieved from online repositories to be used as an outgroup species (Palkopoulou et al. 2018). An overview of the genomic data being used for this study is listed in Table S2.

### 2.2 Mapping of Whole Genome Sequence Data

Short read sequences of 27 Asian elephants and 1 African elephant (Palkopoulou et al. 2018) were obtained and mapped to the mEleMax1 reference genome (genome size = 3.4 Gb) of *E. maximus* , generated by the G10K‐VGP Project (https://www.ncbi.nlm.nih.gov/assembly/GCF_024166365.1). The reference genome was indexed, after which the short reads were mapped using bwa‐mem2 with default settings (v2.2.1, index) (Vasimuddin et al. 2019). Duplicate reads were removed using samblaster (v0.1.26) (Faust and Hall 2014) and followingly mapped read files from the same individual were merged using samtools merge (Danecek et al. 2021). All bamfiles were sorted (samtools sort) and indexed (samtools index) by samtools, and the quality of the mapped reads was assessed by Qualimap (v.2.2.2‐dev) (Okonechnikov et al. 2016). The WGS data resulted in ~21–34× coverage of the genome.

### 2.3 Variant Calling of Single Nucleotide Polymorphisms (SNPs)

Population variant calling was performed on the 27 Asian elephant individuals to detect SNPs, using Freebayes (v1.3.1, ‐‐use‐best‐n‐alleles 4 ‐‐min‐base‐quality 10 ‐‐min‐alternate‐fraction 0.2 ‐‐haplotype‐length 0 ‐‐ploidy 2 ‐‐min‐alternate‐count 2) (Garrison and Marth 2012). Only high‐quality variants with QUAL scores > 20 were included (variants with a 99% chance of having a variant at that specific site) using vcftools (v0.1.16, ‐f ’QUAL > 20’) (Garrison et al. 2022), and indexed using tabix (Tabix v1.17; Li 2011), resulting in an unfiltered set of 22,385,070 variants for the Asian elephant dataset. The quality of the variant calling was assessed using bcftools (v1.9) (Danecek et al. 2021).

Variant calling was performed on the African savanna elephant individual to detect SNPs, but only on the locations within the resulting vcf file of the population variant calling on 27 Asian elephant individuals described hereinabove. For the variant calling on the African savanna elephant we used the same method as for the population variant calling on the Asian elephant individuals.

Both vcf files were merged with vcftools (vcf‐merge). SNPs in this merged file were filtered on quality and minimum and maximum depth using vcftools (v0.1.16, ‐‐minGQ 30 ‐‐minDP 15 ‐ ‐maxDP 60) (Danecek et al. 2015). SNPs with low depth of coverage could be false positives; SNPs with very high depth of coverage could be mapping errors or are likely to be SNPs present in repetitive regions that map to multiple parts of the genome. Furthermore, no missing data was allowed since it might affect Treemix analysis (‐‐max‐missing 1). Following this, the dataset was pruned for linkage disequilibrium using plink (v1.90b3.38, ‐‐indep‐pairwise 50 10 0.1) (Purcell et al. 2007), because Treemix assumes unlinked SNPs. This resulted in a vcf file containing 97,565 SNPs, which was used for the Treemix analysis. For the PCA and ADMIXTURE analysis, the same vcf file was used but then without the African savanna elephant individual included.

### 2.4 PCA and ADMIXTURE

Population genetic structure was assessed with a Principal Component Analysis (PCA) and ADMIXTURE analysis. PCA was performed with plink (v1.90b3.38, ‐‐pca ‐‐chr‐set 28 ‐‐allow‐extra‐chr) (Purcell et al. 2007) to define an overall view of the population structure. ADMIXTURE was inferred with default settings (Alexander et al. 2009) (v1.3.0) to assess the most likely number of clusters formed. No prior information about the genetic structure was assumed, and the number of genetic clusters (K) was predefined from two to five. The PCA and ADMIXTURE output was visualized using ggplot in R (v3.6.2).

### 2.5 TreeMix

A phylogenetic tree based on SNP frequencies was built with TreeMix (v1.13). With PLINK, a .map and .ped file were created using only bi‐allelic SNPs with mac 2 (‐‐mac 2 ‐‐remove‐indels ‐‐max‐alleles 2). Subsequently, we used PLINK to convert genotype data into binary format by generating a .bed file using the ‐‐make‐bed option, with the flags ‐‐allow‐no‐sex to retain individuals without recorded sex and ‐‐allow‐extra‐chr 0 to treat non‐standard chromosomes as unplaced. This binary file was then used to calculate stratified allele frequencies across predefined population groups using the ‐‐freq command in combination with ‐‐within, which references a population assignment file. We also included the ‐‐missing flag to report missingness for each SNP and sample. These frequency files served as the input for conversion to TreeMix format. To generate TreeMix input files, we used Python 2 and the script plink2treemix.py (available at https://bitbucket.org/nygcresearch/treemix/src/master/), which converts PLINK‐formatted files (e.g., .ped/.map or .bed/.bim/.fam) into the allele frequency format required by TreeMix. The script processes population labels to aggregate SNP data into allele count matrices per population, as required by TreeMix for modeling population splits and gene flow. Finally, TreeMix was run with the number of migration edges (−m) set from 0 to 5 (i.e., m ∈ {0, 1, 2, 3, 4, 5}) to model varying levels of historical gene flow between populations. For each migration setting, the inference was replicated three times (i ∈ {1, 2, 3}) using different random seeds to assess the consistency and robustness of the inferred tree topologies and migration events. The African savanna elephant was assigned as the root, and the analysis was done for 500 bootstraps. TreeMix output was visualized in R using custom plotting functions provided in the plotting_funcs.R script included with the TreeMix distribution.

### 2.6 Phylogenetic Analysis

Phylogenetic trees of Asian elephant subpopulations were constructed by the maximum‐likelihood (ML) method of RAxML (v8.2.9), using both autosomes and allosomes since only female elephants were included in the analysis (Alexey et al. 2019). The best‐fitting model of substitutions was identified by jModelTest2 (Darriba et al. 2012). To avoid potential alignment errors caused by segmental duplications or copy number variation, one‐to‐one orthologous genes between the African elephant ( *Loxodonta africana* ) and the nine‐banded armadillo ( *Dasypus novemcinctus* ) were used for the phylogenetic tree. The African elephant was used instead of the Asian elephant because gene orthology information for the Asian elephant was not available. The nine‐banded armadillo was chosen because it is the closest relative to the African bush elephant with one‐to‐one orthologous gene information available. A list of one‐to‐one orthologous genes with corresponding gene names was extracted from ENSEMBL with BioMart (Kinsella et al. 2011). Only orthologous genes with an ortholog confidence of 1 and a conservation score of 100 were included. Orthologous gene regions were extracted by linking the gene name to the gene coordinates of the Asian elephant. This resulted in 5419 orthologous genes. Full gene sequences per orthologous gene were reconstructed using the VCF file created by population variant calling and the reference genome (mElemax1), using GATK (gatk FastaAlternateReferenceMaker ‐R ‐O ‐L ‐V) (Van der Auwera and O'Connor 2020) (individuals included: Table S2). This resulted in a total of 235,037,510 base pairs used for analysis. The SNPs occurring in these regions were extracted and used to make phylogenetic trees; a total of 370,610 SNPs were included. Both supermatrix and supertree approaches were used, with an African savanna elephant ( *Loxodonta africana* , sample ERR2260497), originating from South Africa, as an outgroup (Table S2). A supermatrix approach combines all available sequence data into a single, large alignment for joint phylogenetic analysis, whereas a supertree approach infers a species tree by combining separately inferred gene trees without requiring sequence alignments to be concatenated. The SNPs were previously filtered on depth and quality using vcftools (v0.1.16, ‐‐minQ 30 –minDP 15 –maxDP 60) (Garrison et al. 2022). For each subpopulation, one individual was chosen (eight individuals in total) to represent the subpopulation, based on depth of coverage, female gender, and consistent signal in the PCA and ADMIXTURE analysis. Table S2 shows which individuals were selected for this analysis.

For the supermatrix approach, the concatenated alignment was analyzed under the best fitting substitution model (GTRGAMMA) identified by jModelTest2 (Darriba et al. 2012, ‐g 4 ‐i ‐f ‐AIC ‐BIC ‐a). For the supertree approach, individual gene trees for every elephant‐to‐armadillo orthologous gene were separately inferred, using the GTRGAMMA model. All gene trees, including unresolved trees, were combined in a consensus tree using ASTRAL‐II 5.7.8 (Zhang et al. 2018). Trees were depicted using the software FigTree (v1.4.2) (Rambaut 2010).

### 2.7 Molecular Clock Analysis

Divergence times between subspecies of the Asian elephant were estimated using the Bayesian Phylogenetics and Phylogeography program (BPP) (Yang 2015; Flouri et al. 2018). The method uses the multispecies coalescent model to compare different models of species delimitation (Yang and Rannala 2010; Rannala and Yang 2013) and species phylogeny (Yang and Rannala 2014; Rannala and Yang 2017) in a Bayesian framework, accounting for incomplete lineage sorting due to ancestral polymorphism and gene tree‐species tree discordance. Only one‐to‐one orthologous gene sequences between the African bush elephant and nine‐banded armadillo were used for analysis. For this, we followed the same procedure as described above for the phylogenetic trees. The fixed species tree was based on the phylogenetic tree analysis. One individual of *Loxodonta africana* (ERR2260497) was used as an outgroup (Palkopoulou et al. 2018). For each subspecies: Borneo, Sumatra, Sri Lanka, and Mainland, three individuals were selected to maintain the same number of individuals across the subspecies. For Borneo, Sumatra, and Sri Lanka, this means that all sequenced individuals were incorporated. For the Mainland, the three individuals from Myanmar were chosen since they clustered closely with the Sri Lankan individuals. This choice helps avoid overestimating the divergence time between the Mainland and Sri Lanka. Moreover, in the ADMIXTURE analysis, the individuals from Myanmar showed mostly signs of admixture compared to the other Mainland populations. The population size parameters (θs) are assigned the inverse‐gamma prior IG(3, 0.002), with mean 0.002/(3–1) = 0.001, based on a mutation rate of 2.5 × 10‐8 per site per generation, a generation time of 25 years, and a divergence time between Loxodonta and Elephas of 8 Ma (CI: 23–4 Ma) (Alvarez‐Carretero et al. 2021; Benton et al. 2015). The divergence time at the root of the species tree (τ0) is assigned the inverse gamma prior IG(3, 0.6), with mean 0.32. This was based on half of the branch length between Loxodonta (outgroup) and Elephas on the maximum likelihood tree of the concatenated alignment of orthologous genes (branch length = 0.63946). The other divergence time parameters are specified by the uniform Dirichlet distribution (Yang and Rannala 2010: equation 2). A molecular clock with correlated rates, with a large violation of the molecular clock, was used (v = 0.5). Each analysis is run for 50.000 iterations with a burn‐in of 2000 and run at least twice to confirm consistency between runs. Tau estimates were used to estimate absolute times, using the bppr R package (Angelis and Dos Reis 2015).

### 2.8 Pairwise Sequential Markovian Coalescent (PSMC)

To reconstruct effective population size changes through time, Pairwise Sequentially Markovian Coalescent (PSMC) was used (Li and Durbin 2011). The PSMC method uses heterozygosity patterns across the genome of a single individual and thereby infers the coalescent rate. The proportion of missed heterozygosity was used to scale the PSMC to correct for low coverage. This was obtained by down‐sampling the individual with the highest coverage (ERR2260498) to the coverage of other individuals using SAMtools (v1.12, view ‐b ‐s [ratio]) (Li et al. 2009). All .bam files were indexed using samtools. The .bam files contained only the 10 largest autosomal chromosomes of individuals to cover the majority of the genome and to address computational constraints. The .bam files were filtered for minimum mapping quality of 20 and a minimum base quality of 30, followed by a conversion to fastq files using BCFtools (v1.9, mpileup ‐Q 20 ‐q 30) (Danecek et al. 2021). SAMtools was used to filter for a minimum coverage of 6× and a maximum coverage of two times the average coverage per sample to prevent alignment errors caused by segmental duplications or copy number variation (Alex Buerkle and Gompert 2013) (vcfutils.pl vcf2fq ‐d 6 ‐D ${max_cov} ‐Q 30). The proportion of missing data was calculated after converting fastq data files to fasta format using seqtk v1.3‐r106 (https://github.com/lh3/seqtk, seq ‐a). All samples had below 6% missing data and could be included in PCMC analysis because the proportion was below the limit of 25% missing data (Nadachowska‐Brzyska et al. 2016). PSMC was run in line with other elephant PSMC literature settings (v0.6.4‐r49, ‐N30 ‐t15 ‐r5 ‐p “4 + 252 + 4 + 6”). The proportion of missed heterozygosity per depth of coverage was inferred by visually scaling the heterozygosity false negative rate of the PSMC output to match the original non‐down‐sampled individual. PSMC was run with the settings mentioned above and including the 27 Asian elephant samples in this study and corrected for the proportion of missed heterozygosity. The PSMC output was rescaled to years using a generation time of 25 years and a mutation rate of 2.5 × 10^−8^ per site per generation (Díez‐del‐Molino et al. 2023). The rescaled output was visualized using R (v3.6.2).

### 2.9 Timing of Inbreeding

ROHs were detected utilizing the R package RZooROH (v0.3.1; Bertrand et al. 2019; Druet and Gautier 2017). The set of 97 565 SNPs used for the Treemix analysis served as the input data. RZooRoH employs a hidden Markov model (HMM) to recognize ROHs, also known as homozygous by descent regions (HBD). It facilitates the evaluation of inbreeding across the genome and discerns between recent and ancient inbreeding, as the length of an HBD segment correlates inversely with the recombination rate and the time of the ancestor/inbreeding event (t). A model comprising 14 HBD classes was fitted, with predetermined rates set at {4, 8, 16, 32, 50, 76,100, 150, 250, 500, 1000, 2000 and 5000}. These rates correspond to various age‐related HBD classes and inversely correlate with the expected length of HBD segments (Bertrand et al. 2019). Halving these rates provides an estimate of the time to the common ancestor, offering insights into the timing of population contractions (Bertrand et al. 2019).

## 3 Results

Our dataset included 27 wild born Asian elephant individuals with known provenance and traceable records of origin. Of these, 18 individuals originated from the Mainland (Northern India *n* = 3, Southern India *n* = 3, Myanmar *n* = 3, Thailand *n* = 3, Vietnam *n* = 3, and Peninsular Malaysia *n* = 3); three individuals from Sri Lanka, three individuals from Sumatra, and three individuals from Borneo. Whole genome sequences of these 27 Asian elephants were mapped to the *E. maximus* reference genome (mEleMax1), resulting in an average coverage of ~25× (21.1×–34.1×) per individual (details in Table S2).

### 3.1 SNP Data Reveal a Clear Population Structure Within Asian Elephants

**FIGURE 2:**
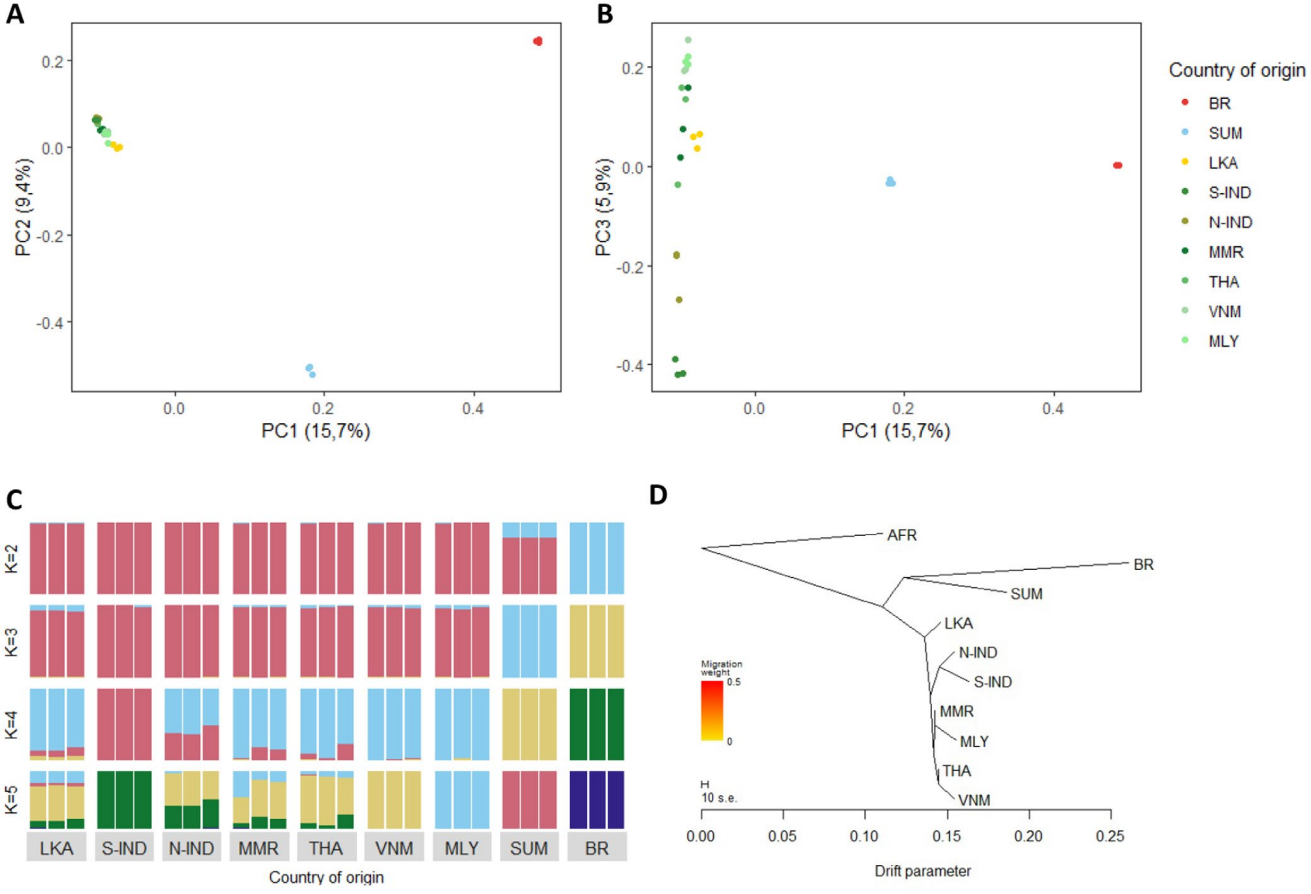
Principal component analysis (A, B), ADMIXTURE analysis (C), and TREEMIX analysis (D) were performed on whole genome SNP data of Asian elephant individuals originating from nine populations: Borneo (BR), Sumatra (SUM), Sri Lanka (LKA), Southern India (S‐IND), Northern India (N‐IND), Myanmar (MMR), Thailand (THA), Vietnam (VNM) and Peninsular Malaysia (MLY) each with *n* = 3.

The Asian elephants form three distinct genetic clusters with a fourth additional separation of Sri Lanka from the Mainland evident in the Principal Component Analysis (PCA, Figure 2). PC1 explains 15.7% of the total variability and separates the Bornean cluster from the other clusters, with the Mainland and Sri Lankan clusters on the other extreme and the Sumatran cluster in the middle. On PC2, 9.4% of the total variability is explained, and the Sumatran cluster is separated from the other clusters. Here we observe the Sumatran cluster on the most extreme, whereas the Mainland and Sri Lankan clusters are positioned closer to the Bornean cluster. On both PC1 and PC2, the Mainland and Sri Lankan clusters are positioned close to each other. However, PC3 (5.9%) reveals genetic structure within the Mainland cluster, albeit less pronounced than between the Mainland cluster and the Bornean or Sumatran clusters. The PC3 axis closely aligns with the natural geographic distribution spanning from Southern to Northern Indian elephants, via Myanmar and Thailand individuals, to Vietnam and Peninsular Malaysia at the opposite end of the distribution. One individual from Thailand does not fit the expected pattern, as it is situated between Northern Indian and Myanmar individuals, whereas the other two Thai individuals, as expected, are situated between Myanmar and Vietnamese individuals. Surprisingly, the Sri Lankan individuals align most closely with the Myanmar individuals.

Admixture analyses corroborate the population structure observed in the PCA results. With K = 2, the Bornean individuals appear as a distinct cluster separate from the other Asian elephant individuals, while Sumatran elephants exhibit genomic signatures of both clusters. With K = 3, the Sumatran individuals form a distinct cluster. At K = 4, Southern Indian elephants are separated from those in Vietnam and Peninsular Malaysia while elephants from countries in between exhibit decreasing admixture proportions based on geography. As observed in PC3, elephants from Sri Lanka display admixture proportions akin to those of the Myanmar individuals. At K = 5, Vietnam and Peninsular Malaysia individuals separate from each other, while Sri Lankan individuals still show some admixture proportions with Sumatran individuals. Furthermore, across various K values, Sri Lankan individuals and those from mainland populations are consistently positioned within the same genetic cluster or exhibit some semblance of the same cluster. Examining the cross‐validation (CV) values, K = 2 emerges as the most significant number of populations.

A maximum likelihood tree constructed with TREEMIX, based on allele frequencies, is consistent with both the supermatrix (Figure S3) and supertree (Figure S4) built from the full sequence of one‐to‐one orthologous genes between African elephants and nine‐banded armadillo. Nine‐banded armadillo was chosen because it is the closest relative to African bush elephant with one‐to‐one orthologous genes information available. For TREEMIX analysis, the African savanna elephant has been used as an outgroup. In Figure 2D, the tree with no migration edges is depicted. Figure S5 shows the TREEMIX results for migration edges 0–5. This TREEMIX result shows that larger drift effects are apparent within the Bornean elephants compared to the other Asian elephant populations.

### 3.2 Molecular Clock Estimates Reveal Recent Divergence Within Asian Elephants

**FIGURE 3:**
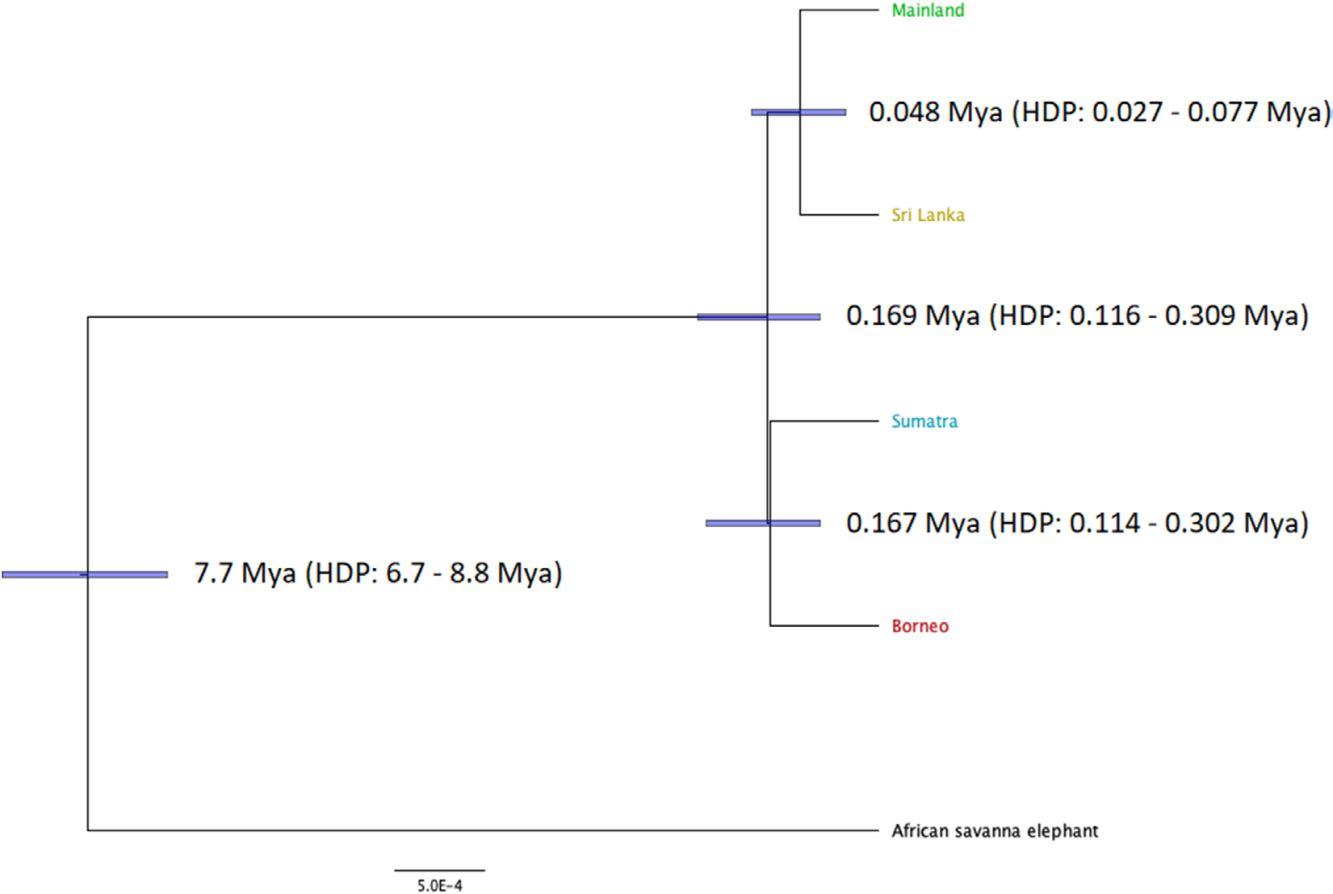
Phylogenetic tree with divergence time estimated by BPP based on the full sequence of 5419 one‐to‐one orthologous genes between African elephants and armadillos. For the included Asian elephant individuals, refer to Table S2. One sequence of African savanna elephant was used as an outgroup. HPD, highest posterior density interval; Mya, million years ago.

Divergence times between the Asian elephant subspecies found in the phylogenetic analyses were estimated with the program BPP (Figure 3) using a calibration time of 7.7 mya for the basal African/Asian split. Divergence times within the Asian elephant occurred relatively recently, namely between 169,000 and 48,000 years ago. First, the Asian elephant common ancestor diverged into a Mainland‐Sri Lankan ancestor and a Bornean‐Sumatran ancestor. This split took place ca. 169,000 years ago. Soon after this split, the Borneo and Sumatra elephants diverged from each other. The most recent split within the Asian elephant species is between the Mainland and Sri Lankan populations. This split occurred around 48,000 years ago (HDP: 27,000–77,000 years).

### 3.3 Historical Population Analyses Show Recent Bottlenecks

**FIGURE 4:**
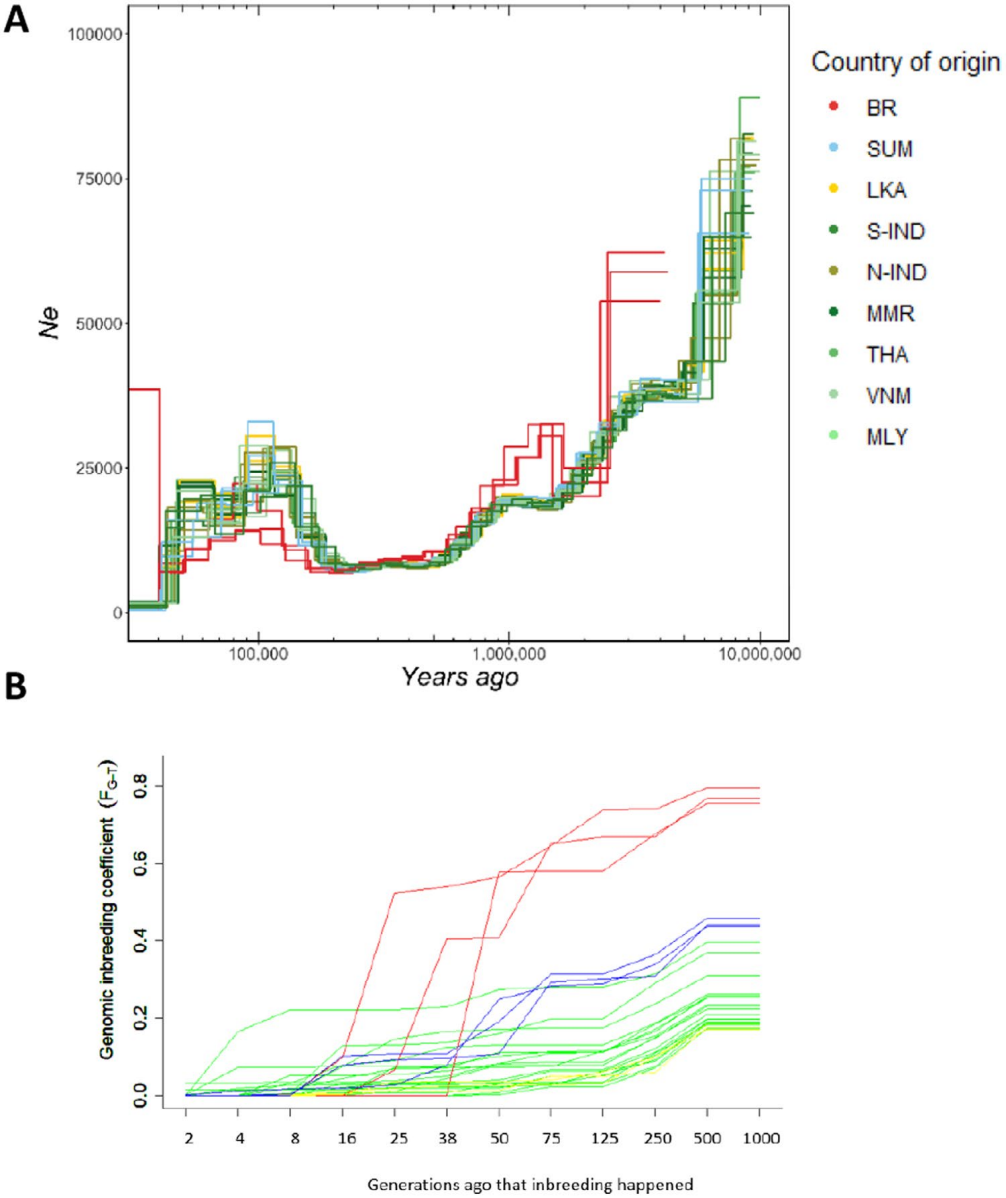
(A) Historical effective population sizes (Ne) of nine Asian elephant populations estimated with PSMC with a generation time (g) of 25 years and a mutation rate (μ) of 2.5 × 10^−8^ per site per generation. The *x*‐axis is logarithmic transformed. (B) The accumulation of inbreeding (FROH) per individual over different generations back in time is represented by the following colors: Red lines for Bornean individuals; blue lines for Sumatran individuals; yellow lines for Sri Lankan individuals; green lines for Mainland individuals.

This relatively recent divergence among Asian elephants is supported by reconstructing historical effective population sizes using PSMC. All inferences of effective population sizes for various individuals show a similar trajectory over time (Figure 4A). The trajectories of the Bornean individuals appear somewhat skewed, likely influenced by the known high level of homozygosity within these individuals, as previously reported (Palkopoulou et al. 2018). The evolutionary history reveals several bottlenecks occurring between 500,000 and 10 million years ago. Around 200,000 years ago, Asian elephant populations started to experience a slight recovery and divergence from each other. Populations began to decrease again around 100,000 years ago. The slight deviations in the trajectories observed around 200,000 years ago align with the estimated divergence time for the Asian elephant ancestor, which is approx. 169,000 years.

Since PSMC is primarily reliable for detecting ancient bottlenecks rather than indicating more recent ones, we ran RZooROH to analyze recent inbreeding events. This analysis with RZooROH (Figure 4B) across Asian elephant populations reveals that all populations experienced an increase in genomic inbreeding between 250 and 500 generations ago. Moreover, two populations experienced severe bottlenecks more recently. The Sumatran elephant experienced an increase in genomic inbreeding between 50 and 75 generations ago, while the Bornean elephant experienced a significant rise in genomic inbreeding between 8 and 38 generations ago.

## 4 Discussion

In this study, we present WGS data covering nearly the entire distribution range of Asian elephants. We aimed to validate the current designation of Asian elephant subspecies and address existing taxonomic ambiguities, particularly focusing on the Bornean and Sri Lankan elephants, through the use of new genomic data. Our results demonstrate (1) clear population structure among Asian elephants. We identify three distinct genetic clusters: Borneo, Sumatra, and Mainland, along with a fourth separation of Sri Lanka from the Mainland. Additionally, we found (2) relatively recent splits between the different genetic clusters, ranging from 170,000–50,000 years ago. Furthermore, we identified (3) several ancient bottlenecks occurring between 500,000 and 10 million years ago. The Sumatran and Bornean elephants experienced recent severe bottlenecks that are not evident in the other Asian elephant populations. Taking it all together, our study confirms the current designation of Asian elephant subspecies based on WGS data. This study is the first to validate the subspecies status of the Sumatran elephant population using WGS data.

### 4.1 Genomic Data Supports Subspecies Status for Bornean Elephants

Although Bornean elephants appear as a distinct cluster in this study, questions about their origin remain. Two hypotheses have been previously proposed. One suggests that Bornean elephants are native and naturally dispersed to Borneo during glaciations in the Pleistocene, possibly originating from India, Sumatra, or Peninsular Malaysia (Fernando et al. 2003; Sharma et al. 2018). The other hypothesis states that elephants were introduced by humans between the 16th and 18th centuries. This hypothesis is based on historical records suggesting that the current population descends from a domesticated herd that formerly existed on Sulu Island, Philippines, and was introduced to Eastern Sabah by the Sultan of Sulu (Cranbrook et al. 2008; Shim 2003; Sharma et al. 2018). The origin of the elephants that formed this domesticated herd on Sulu Island is also not clear, with both Java and the Mainland being proposed as possible sources (Sharma et al. 2018). Our results do not support the Mainland as a source population under this assumption. For long, the fact that no fossils of *Elephas maximus* were found on Borneo has been used as support for the introduction hypothesis. However, a newly recognized Asian elephant ( *Elephas maximus* ) specimen re‐discovered among the animal remains excavated by the Harrissons at West Mouth (Niah Cave) in 1958 contradicts this common belief (Tshen 2022).

This finding implies that there have been elephants on Borneo since the late Pleistocene. In addition, the fact that only one unique mtDNA haplotype has been found in Bornean elephants could suggest a native origin of Bornean elephants (Fernando et al. 2003). However, it could also be indicative of a small founder population, thus supporting the introduction hypothesis. If we assume that the Bornean elephants are native to the island, our results support Sumatra as the source population and reject India and Peninsular Malaysia as the source populations, as previously suggested (Sharma et al. 2018). We estimate the divergence time between the Bornean and Sumatran elephant to be 167,000 years ago (HDP: 114,000–302,000). This aligns with the estimated divergence time for the Bornean elephant based on mtDNA analysis, which is 300,000 years (Fernando et al. 2003). Glaciations during the Pleistocene took place between 2.6 million and 20,000 years ago. This suggests that the estimated divergence times based on mtDNA and nuclear DNA could support colonization during the Pleistocene and the hypothesis that Bornean elephants are native to the island. The estimated divergence time is similar to that of other species living both on Borneo and Sumatra. For example, the separation between the Sumatran rhinoceros living on Borneo ( *Dicerorhinus sumatrensis harrissoni* ) and Sumatra ( *Dicerorhinus sumatrensis sumatrensis* ) occurred approximately 300,000 years ago based on mtDNA analysis (von Seth et al. 2021). Similarly, the split between the Bornean orangutan ( *Pongo pygmaeus* ) and the Sumatran orangutan ( *Pongo abelii* ) is estimated to have occurred between 300,000 and 400,000 years ago (Locke et al. 2011; Mailund et al. 2011).

If we assume the introduction hypothesis with Java as the source population, the divergence time between Bornean and Sumatran elephants should reflect the divergence time between Sumatran elephants and the now extinct Javan elephants. Other estimates of divergence times between Sumatran and Javan species are not abundant, making comparisons difficult. However, our results support severe bottlenecks for the Bornean elephant population occurring between 8 and 64 generations ago. With a generation time of 25 years, this could reflect the bottleneck caused by the introduction of some elephants on the island of Borneo between the 16th and 18th centuries. As mentioned, these elephants originated from a domestic herd, implying that right before their introduction to Borneo, this population experienced another bottleneck, which is in line with our results. However, estimates on when this domestic herd was founded are lacking. Contrarily, these severe bottlenecks could also reflect the anthropogenic impact on this native population. In such a case, it would be expected that these severe bottlenecks would be apparent in other Asian elephant populations too, which is not consistent with observations. In conclusion, with our results we cannot draw a definite conclusion on which of the two previously proposed hypotheses about the origin of the Bornean elephant is true. However, our results show that under the native origin hypothesis, Sumatra seems to be the source population, while under the introduction hypothesis, we reject Mainland Asia as the source population for the domesticated herd from which individuals were introduced to Borneo. This leaves Java as the only remaining option from what has been previously suggested. Moreover, our results do not reject this assumption. Further research should focus on retrieving genomic data from the now extinct Javan elephant.

Based on our genomic results, we support the recently assigned subspecies status for the Bornean elephant (IUCN 2024). When the Bornean elephant is native to the island, it represents a unique lineage of Asian elephants that should be preserved. If the Bornean elephant is considered a feral population, it may harbor genetic diversity once present in the now extinct Javan elephant population. From a genomic perspective in both scenarios, it is worthwhile to manage the Bornean elephant as a separate unit. In that regard, we support recognizing the Bornean elephant as an Asian elephant subspecies to prevent the loss of unique genetic diversity. Further research should provide more insight into the extent of this unique genetic diversity within this population, particularly as it may result from local adaptation to the distinct island environments. In addition, further research should examine the genetic viability of the Bornean elephant population.

### 4.2 Ambiguities Surrounding Status of Sri Lankan Elephant Remain

Questions also remain about the subspecies status for the Sri Lankan elephant population, *Elephas maximus maximus* . Molecular genetic studies using mtDNA indicate no major genetic differentiation between the Sri Lankan elephant and the mainland species (Hartl et al. 1996; Fernando et al. 2000; Fleischer et al. 2001; Sukumar 2003). However, it is important to note that the Sri Lankan population is harboring mtDNA haplotypes that are also present in northern Indian and Myanmar populations, but are absent in southern Indian populations, which are geographically more nearby (Vidya et al. 2009). Our genome analysis results corroborate previous mtDNA studies, indicating no significant genetic differentiation between the Sri Lankan population and the mainland population. We estimate the divergence time between the mainland population and the Sri Lankan population at 48,000 years. Till 10,000–15,000 years ago, Sri Lanka and the mainland were connected via a land bridge called “Adam's Bridge” or “Rama's Bridge” which connected Sri Lanka with the southern tip of India (Jacob 1949).

While our estimated divergence time is in line with that observation and points towards a dispersal of elephants from the Southern tip of India to Sri Lanka, the fact that the Sri Lankan individuals in our analysis cluster with the Myanmar individuals instead of the Southern Indian individuals may be surprising based on geographic distance, but less so in light of historical trade routes. Sri Lanka was a center of domestic elephant trade within the region, with exportations and importations of elephants recorded historically (Fernando et al. 2000). However, it has been claimed that these domestic elephants were unlikely to be introduced into the wild (Fernando et al. 2000). Moreover, the individuals that we used as a reference for the Sri Lankan elephant population in this study are elephants that have been traded to the European continent at some point, so this raises questions about whether these individuals or their ancestors were part of this trade network and can be used as a proxy for the original genetic diversity within the wild Sri Lankan population. Future nuclear genomic research with a larger sample size for the Sri Lankan elephant population should give more insight into the history of this population and thereby its taxonomic state.

In our study, the majority of the Asian elephant individuals included, namely 23 out of 27, are born in the wild but have lived or are currently living in European zoos. For these individuals, we based their country of origin on documentation from decades ago. Nowadays, it is hard to check the accuracy of this documentation because zoo employees who were working at the time of the importations have retired or passed away. This means that we have to account for some bias in the assumed country of origin. However, our results show clear structure, and almost all the assumed countries of origin are supported by our analysis. There is only one individual with assumed origin Thailand that can be seen as an outlier compared to the other two individuals with the same origin (Figure 2B). Furthermore, for three (Borneo, Sri Lanka and Sumatra) of the eight sample locations, two out of the three individuals were transported to the European continent on the exact same day and were coming from the same source location, so this means that there can be some relatedness between these individuals, which could influence our analyses. Unfortunately, data on the identity of their parents is missing. However, for these three locations, there is still one individual that is not related to the two others, and the population structure that we have shown seems to be robust.

### 4.3 Wide Genomic Variety in European Ex Situ Population

Our dataset shows that the founders of the EAZA Ex Situ Program (EEP) population of Asian elephants harbor a wide genetic diversity with representatives of all four indicated genetic clusters. In terms of conservation, this can be seen as a sign of genetic health and a comprehensive suite of diversity. Yet, it also implies that genetic management based on genomic data is needed to maintain the full spectrum of diversity present and thereby optimize the conservation value of this ex‐situ population. Following the SLOSS debate, one can argue to manage the different clusters separately to avoid, for example, outbreeding depression and maintain genetic diversity between clusters or to manage the different clusters as one big population to avoid loss of genetic diversity within clusters by excessive inbreeding and genetic drift. Further research into the Asian elephant relationships across their geographic range and the genetic health of the different clusters can help define conservation priorities and thereby determine the optimal management for this ex‐situ population.

In summary, our study provides the most comprehensive whole‐genome dataset to date covering the full distribution range of Asian elephants, offering novel insights into population structure, evolutionary divergence times, and experienced population bottlenecks. We confirm the genetic distinctiveness of the four recognized Asian elephant subspecies, including the first genomic validation of the Sumatran elephant's status and genomic support for treating Bornean elephants as a separate subspecies. While the origin of Bornean elephants remains unresolved, our findings refine the existing hypotheses and suggest Sumatra or Java as plausible source populations, depending on whether the population is native or introduced. We also highlight the need for further genomic research into the Sri Lankan elephant population to clarify its taxonomic placement. From a conservation perspective, our findings are critical for both in situ and ex situ strategies: the identification of distinct genetic clusters can help prioritize conservation units and guide local management plans aimed at preserving evolutionary potential in the wild, while the genomic characterization of the EAZA Ex situ Program population reveals a broad representation of this diversity. This underscores the importance of genomics‐informed breeding strategies for this ex situ population to maintain both intra‐ and inter‐population variation, avoid outbreeding depression, and support potential future reintroduction programs. Continued genomic research across the Asian elephant's range is essential to define conservation priorities and ensure the long‐term persistence of this endangered species.

## Author Contributions

**Jeroen Kappelhof:** conceptualization (lead), data curation (lead), formal analysis (lead), funding acquisition (supporting), investigation (lead), methodology (equal), project administration (supporting), validation (equal), visualization (equal), writing – original draft (lead), writing – review and editing (lead). **Emma Diepeveen:** data curation (equal), formal analysis (equal), methodology (equal), visualization (equal), writing – review and editing (equal). **Martijn F. L. Derks:** data curation (supporting), formal analysis (supporting), validation (supporting), writing – review and editing (supporting). **Ole Madsen:** conceptualization (supporting), methodology (supporting), validation (supporting), writing – review and editing (equal). **Rebekah Rogers:** resources (equal), writing – review and editing (equal). **Benoit Goossens:** writing – review and editing (equal). **Reeta Sharma:** validation (equal), writing – review and editing (equal). **Martien A. M. Groenen:** conceptualization (equal), methodology (equal), project administration (equal), supervision (supporting), validation (equal), writing – review and editing (equal). **Jack J. Windig:** conceptualization (equal), methodology (equal), supervision (supporting), validation (equal), writing – review and editing (equal). **Mirte Bosse:** conceptualization (equal), formal analysis (supporting), funding acquisition (lead), methodology (equal), project administration (lead), supervision (lead), validation (equal), visualization (supporting), writing – original draft (supporting), writing – review and editing (equal).

## Conflicts of Interest

Jeroen Kappelhof is the Asian elephant EEP Coordinator.

## Supporting information


**Data S1:** ece372019‐sup‐0001‐Supinfo.docx.

## Data Availability

The raw data that support the findings of this study are available via https://public.anunna.wur.nl/ABGC/Elephants_fastq/.
