# Supplementary material for: Genomics Reveals Distinct Evolutionary Lineages in Asian Elephants
Source: Ecol Evol. 2025 Aug 18;15(8):e72019. doi: 10.1002/ece3.72019 (PMC12361634; doi:10.1002/ece3.72019)
Supplement: Supplementary file 1 — Data S1: ece372019‐sup‐0001‐Supinfo.docx. [file ECE3-15-e72019-s001.docx]

## Supplementary information

Table S1. Overview of software used to analyze whole genome data.

| Name software | Source | Identifier |
| --- | --- | --- |
| Vcftools | Danecek et al., 2011 | [https://github.com/vcftools/vcftools](about:blank) |
| Bcftools | Danecek et al., 2015 | http://www.htslib.org/ |
| Bedtools | Quilan & Hall, 2010 | [https://github.com/arq5x/bedtools2](about:blank) |
| PSMC | Li & Durbin, 2009 | [https://github.com/lh3/psmc](about:blank) |
| Plink | Purcell et al., 2007 | https://www.cog-genomics.org/plink2/ |
| Vcf2phylip | Ortiz, 2019 | [https://github.com/edgardomortiz/vcf2phylip](about:blank) |
| ADMIXTURE | Alexander et al, 2009 | [https://github.com/NovembreLab/admixture](about:blank) |
| ASTRAL | Zhang et al., 2018 | [https://github.com/smirarab/ASTRAL](about:blank) |
| jModelTest2 | Darriba et al., 2012 | [https://github.com/ddarriba/jmodeltest2](about:blank) |
| RAxML | Alexey et al., 2019 | [https://github.com/amkozlov/raxml-ng](about:blank) |
| Figtree | Rambout, 2010 | [https://github.com/rambaut/figtree/releases](about:blank) |
| Samtools | Li et al., 2009 | [http://www.htslib.org/](about:blank) |
| GATK | Van der Auwera & O'Connor, 2020 | [https://github.com/broadinstitute/gatk](about:blank) |
| BPP | Flouri et al., 2019 | [https://github.com/bpp/bpp](about:blank) |

Table S2**.** Overview of sample origin. NG=Novogene. BTS=BGI TECH SOLUTIONS

| **Sample ID** | **House name** | **Country of origin** | **Sex**  **(M/F)** | **Date of entry** | **Sampled by** | **Coverage (X)** | **Phylogeny tree** | **BPP** |
| --- | --- | --- | --- | --- | --- | --- | --- | --- |
| SRR2008170 | Parvathy | India | F | - | Tollis et al., 2021 | 25,63 |  |  |
| FCHLK3MDSX383 | Praya | India | F | 30-12-1979 | This study (BTS) | 22,42 |  |  |
| SRR2009586 | Asha | India | F | - | Tollis et al., 2021 | 28,72 |  |  |
| EKDN230016703 | Kanaudi | India | F | 1-4-1974 | This study (NG) | 24,04 | X |  |
| EKDN230016705 | Tina | India | F | 1-1-1961 | This study (NG) | 26,47 | X |  |
| EKDN230016707 | Mogli | India | F | 1-4-1974 | This study (NG) | 22,67 |  |  |
| ERR2260498 | Moola | Myanmar | F | - | Tollis et al., 2021 | 34,19 |  | X |
| EKDN230016701 | Ankhor | Myanmar | M | 15-6-1989 | This study (NG) | 22,69 |  | X |
| EKDN230016710 | Kewa | Myanmar | F | 13-9-1990 | This study (NG) | 28,37 | X | X |
| EKDN230016698 | Tonsak | Thailand | M | 27-11-2001 | This study (NG) | 22,59 |  |  |
| EKDN230016700 | Saonoi | Thailand | F | 07-04-2004 | This study (NG) | 21,13 |  |  |
| FCHLKFHDSX377 | Douanita | Vietnam | F | 10-7-1988 | This study (BTS) | 27,10 | X |  |
| FCHLKFHDSX376 | Delhi | Vietnam | F | 17-6-1987 | This study (BTS) | 22,43 |  |  |
| EKDN230016695 | Lai Sinh | Vietnam | F | 30-8-1994 | This study (NG) | 26,71 |  |  |
| EKDN230016699 | Janitha | Sri Lanka | F | 7-10-2012 | This study (NG) | 22,42 |  | X |
| EKDN230016704 | Thamara | Sri Lanka | F | 7-10-2012 | This study (NG) | 26,56 | X | X |
| EKDN230016706 | Ceyla Himali | Sri Lanka | F | 1-3-1976 | This study (NG) | 22,23 |  | X |
| FCHLKFHDSX372 | Valentino | Sumatra | M | 9-2-2004 | This study (BTS) | 24,78 |  | X |
| FCHLKFHDSX375 | Cynthia | Sumatra | F | 12-1-1995 | This study (BTS) | 23,81 |  | X |
| FCHLKFHDSX373 | Nova | Sumatra | F | 26-11-1993 | This study (BTS) | 29,81 | X | X |
| FCHLKFHDSX379 | Manari | Borneo | F | 26-8-1998 | This study (BTS) | 22,39 |  | X |
| FCHLK3MDSX382 | Sayang | Borneo | F | 26-8-1998 | This study (BTS) | 22,65 |  | X |
| EKDN230016694 | Pang Pha | Thailand | F | 30-9-1987 | This study (NG) | 27,26 | X |  |
| ERR2260499 | Chendra | Borneo | F | - | Tollis et al., 2021 | 27,91 | X | X |
| EKDN240012680 | Gambir | Malaysia | F | 7-10-1991 | This study (NG) | 23,20 |  |  |
| EKDN240012683 | Sri Nandong | Malaysia | F | 16-1-1989 | This study (NG) | 22,51 |  |  |
| EKDN240012681 | Tun | Malaysia | F | 7-10-1991 | This study (NG) | 22,75 |  |  |
| ERR2260497 | Swazi | South Africa | F | - | Palkopoulou et al., 2018 | 30 | X | X |

Maximum likelihood (ML) phylogenetic trees based on 5,419 one-to-one orthologous genes between African elephants and armadillos inferred with the supermatrix (Figure S3) and supertree (Figure S4) approaches show that the Asian elephant common ancestor diverged into ancestral lineages consisting of a Bornean-Sumatran clade and a Mainland-Sri Lanka clade. Within the Mainland-Sri Lanka clade, the Sri Lankan elephants is the outgroup all the different Mainland populations.


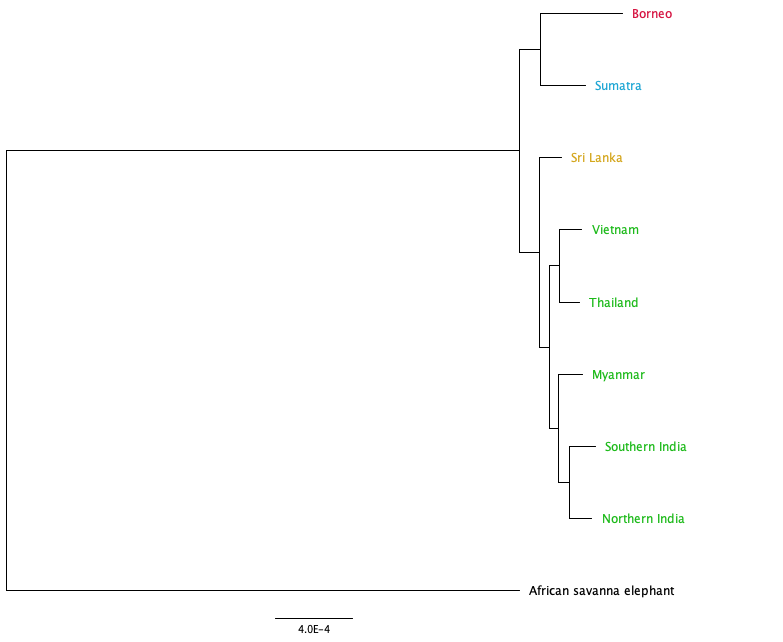


Figure S3. Maximum likelihood phylogenetic supermatrix tree based on the full DNA sequence of 5,419 one-to-one orthologous genes between African elephants and armadillos.


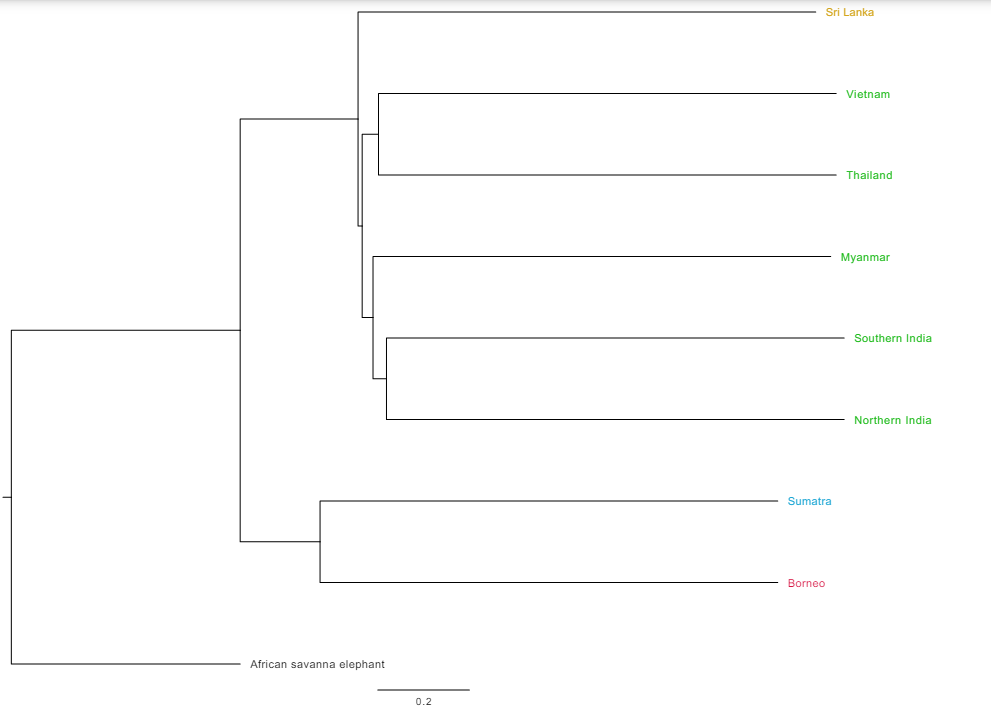


Figure S4. Maximum likelihood phylogenetic supertree based on the full DNA sequence of 5,419 one-to-one orthologous genes between African elephants and armadillos.


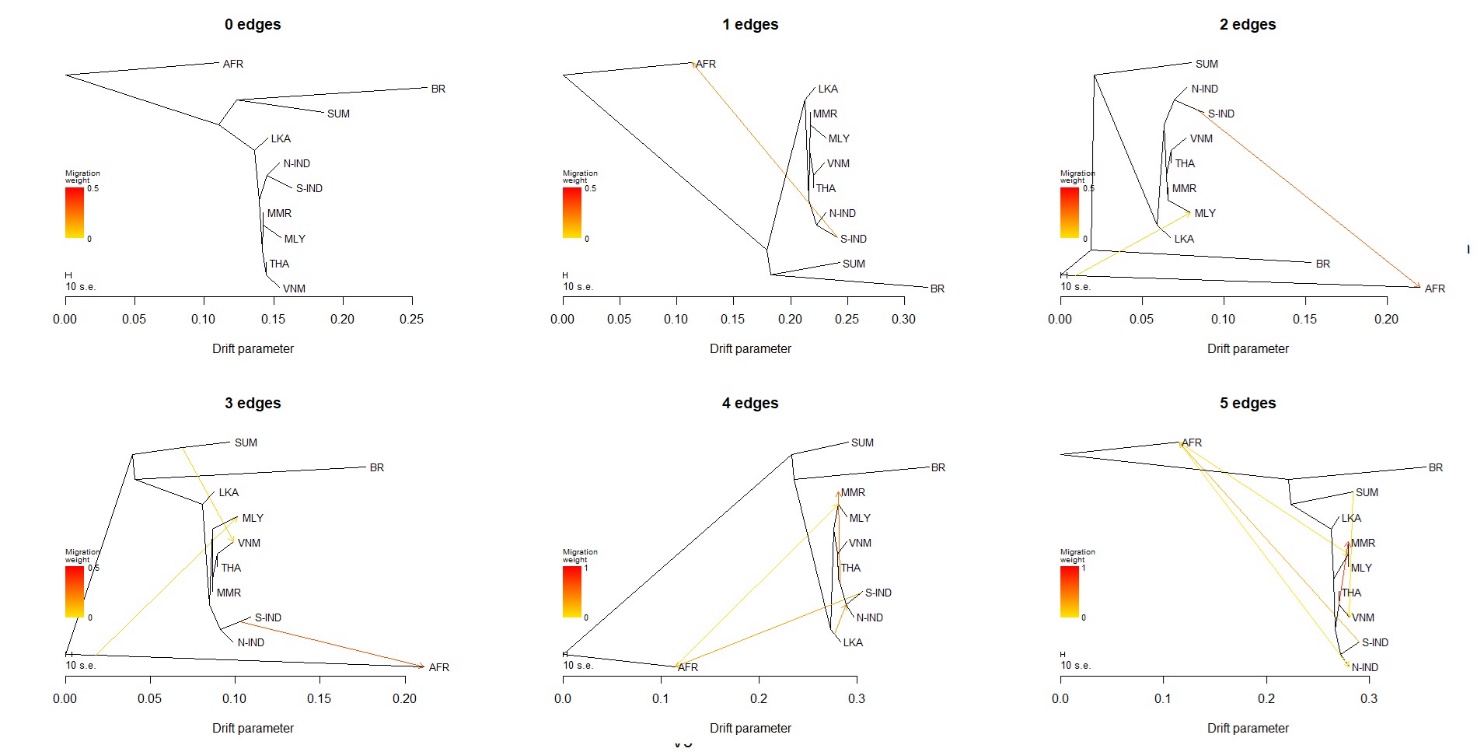


Figure S5. Treemix plot showing the estimated migration edges between populations, with migration events ranging from 0 to 5. The edges represent the inferred gene flow between different groups, highlighting the complexity of historical population interactions and migration patterns.
